# Investigating the Composition and Conductance Distributions on Highly GeSi Mixed Quantum Dots and Inside Oxidation Problem

**DOI:** 10.1186/s11671-015-1185-8

**Published:** 2015-12-09

**Authors:** F. F. Ye, Y. J. Ma, Y. Lv, Z. M. Jiang, X. J. Yang

**Affiliations:** State Key Laboratory of Surface Physics and Collaborative Innovation Center of Advanced Microstructures, Fudan University, Shanghai, 200433 China

**Keywords:** GeSi quantum dots, Conductive atomic force microscopy, Composition distribution, Conductance distribution, Selective chemical etching

## Abstract

With the help of a nanoscale trench, the composition and conductance distributions of single GeSi quantum dots (QDs) are obtained by conductive atomic force microscopy combined with selective chemical etching. However, the obtained composition and current distributions are unwonted and inconsistent on the QDs grown at 680 °C. With a series of confirmatory experiments, it is suggested that a thick oxide layer is formed and remains on the QDs’ surface after etching. Though this selective chemical etching has already been widely applied to investigate the composition distribution of GeSi nanostructures, the oxidation problem has not been concerned yet. Our results indicate that the oxidation problem could not be ignored on highly GeSi mixed QDs. After removing the oxide layer, the composition and conductance distributions as well as their correlation are obtained. The results suggest that QDs’ current distribution is mainly determined by the topographic shape, while the absolute current values are influenced by the Ge/Si contents.

## Background

Self-assembled GeSi quantum dots (QDs) have received great interests for their promising applications in optoelectronics and quantum information technology due to their unique properties and compatibility with the Si technology [1–4]. In the past years, the composition distributions of GeSi QDs have been greatly concerned as it determines the QDs’ electronic structure together with their size and shape. Many methods have been applied to get the composition distributions of GeSi QDs [5], including anomalous X-ray scattering [6–8], cross-sectional transmission electron microscopy (TEM) [9–11], Raman spectroscopy [12], and atomic force microscopy (AFM) combined with selective chemical etching [11, 13–19]. Among these methods, AFM combined with selective etching in a NHH solution (28 % NH_4_OH:31 % H_2_O_2_ = 1:1), which can selectively remove GeSi alloys with the etching rate approximately exponential with the Ge content [20, 21], has been particularly applied due to its simplicity and effectiveness. Recently, three-dimensional (3D) composition profiles of GeSi QDs have been achieved by AFM imaging, the same QDs after NHH etching [22].

On the other hand, the QDs’ electrical properties have also attracted a large number of researches since they are essentially important for the practical applications. To get the QDs’ electrical properties, measurement on single QDs is particularly important as it can exclude the averaging effect. Therefore in recent years, scanning probe microscopy (SPM)-based techniques have been attempted to investigate the electrical properties on single QDs [23–32]. Among these techniques, conductive atomic force microscopy (CAFM) is mostly often applied. The conductive properties of individual InAs, InGaAs, and GeSi QDs have already been achieved by CAFM [27–32]. However, the combined studies on the QDs’ composition distribution and their electrical properties are still lacking, and thus the correlations between these two characteristics are not clear yet.

In this paper, with the help of a nanoscale trench, the 3D composition and conductance distributions are simultaneously measured on same single GeSi QDs by combining CAFM measurement with NHH etching. The results present that the highly GeSi mixed QDs (grown at 680 °C) exhibit extremely poor conductance after 2-min NHH etching, which can be recovered after the subsequent NHH etching processes. With further confirmatory experiments, it is predicted that a thick oxide layer is formed and remains on these QDs’ surface after NHH etching. This oxidation problem has never been mentioned in previous experiments with similar etching method, probably due to the following: conductive properties were not concerned in those cases or the dealt QDs were not highly GeSi mixed. Therefore, our experimental findings should be useful to make clear the NHH etching process of highly GeSi mixed QDs, as well as to get the exact composition and conductive properties of those kinds of QDs.

## Methods

The GeSi QDs studied here were grown on a *p*-type Si(001) wafer (1 ~ 10 Ω cm) by solid source molecular beam epitaxy. The Si substrates were chemically cleaned using the Shiraki method, and the protective oxide layer was removed by heating the substrate at 1000 °C for 10 min inside the growth chamber. The substrate temperature was then lowered to 640 °C, and a 64-nm Si buffer layer was deposited at a growth rate of 0.08 nm/s, followed by a 12 ML Ge layer deposition at the temperature of 680 °C with a growth rate of 0.01 nm/s. The selective chemical etching was performed by dipping the sample in a fresh NHH solution and rinsing in flowing deionized water. Before each NHH etching process, the sample was dipped in a 10 % HF solution for 30 s to remove the native oxide layer and the same as the original sample.

The topography and current measurements were carried out with a commercial AFM equipment (Multi-Mode V, Bruker) at room temperature. The topographic images of GeSi QDs were obtained by AFM in tapping mode, while their conductive properties were measured by CAFM in contact mode. Pt-coated Si tips were used in CAFM measurements, and the bias voltage was applied to the substrate while the tip was grounded. To reduce the influence of local anode oxidation, the current images were measured at negative sample biases and all experiments were performed in a flowing nitrogen atmosphere. To realize the measurements on the same QDs before and after etching, a nanoscale trench with more than 20 nm in depth was made by the AFM tip during scanning, as introduced in our previous work [33]. As the bottom of the trench reaches the pure Si buffer layer, it can hardly be etched in NHH solution since the etching rate decreases to be smaller than 0.01 nm/min for pure Si [20, 21] and hence can act as a better height benchmark than the wetting layer (WL).

## Results and Discussion

**Fig. 1:**
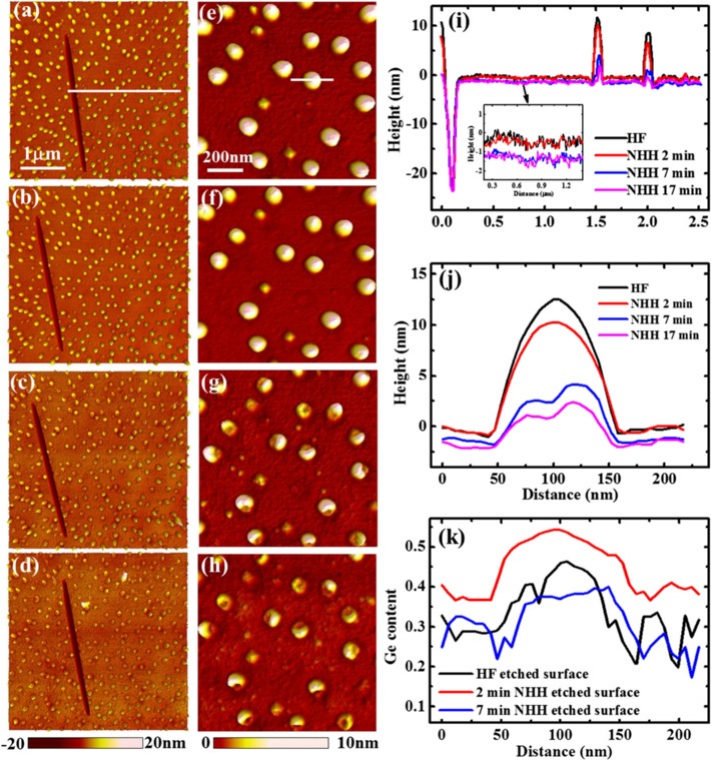
The topography images of the scratched trench and the zoomed area before (**a**, **e**) and after NHH etching for totally 2 (**b**, **f**), 7 (**c**, **g**), and 17 min (**d**, **h**), respectively. The height profiles along the marked line in (**a**–**d**) are shown in (**i**). The zoomed plot in the inset of (**i**) clearly shows the decline of the wetting layer by each etching process. The height profiles along the marked line in (**e**–**h**) are shown in (**j**) and the deduced composition profiles are plotted in (**k**)

The representative large-scale topography images measured on the same area before and after three successional NHH etching processes of 2, 5, and 10 min are shown in Fig. 1a–d, respectively. The height profiles along the marked line across the trench are plotted in Fig. 1i, which have been aligned according to the bottom of the trench. From the zoomed plot, it can be seen that the WL is almost not changed after 2-min NHH etching. But after the subsequent 5-min NHH etching, about a 1 nm of the WL is etched away. The results that the beneath WL has larger etching rate (higher Ge content) than the top one is somewhat surprised because in common sense Ge is enriched at the top of the WL.

Similar unwonted result is obtained on QDs. The topography images of same QDs before and after the same etching processes as above are presented in Fig. 1e–h, respectively. The height profiles of the same QD along the same line are given in Fig. 1j, which are aligned by considering the etched height of the WL in each etching process. It can be seen that, after the initial 2-min NHH etching, only a thin layer on the QD’s top center is removed. To the contrary, a large amount of the QD is etched away after the subsequent 5-min NHH etching. By subtracting the height line after etching from that before etching, the etched height and hence the etching rate are obtained. Previous studies found that the NHH etching rate (*r*) increased with the Ge content (*x*) exponentially [20, 21], which could be written as follows: *r* = *ae*^*bx*^ (with *a* = (7.3 ± 0.4) × 10^−4^ nm/min and *b* = 14.4 ± 0.1) [21]. Using this equation, the etching rate can be converted to the Ge content. The composition profiles along the marked line are plotted in Fig. 1k. It can be observed that the Ge content on the QD’s top layer (before NHH etching) is about 45 %, but it increases to about 55 % at underneath region of the QD (2-min NHH-etched surface). These results suggest that Ge is not enriched in QDs’ top layer but in the beneath region instead, which again violates the common sense as reported in previous literatures [6–19].

**Fig. 2:**
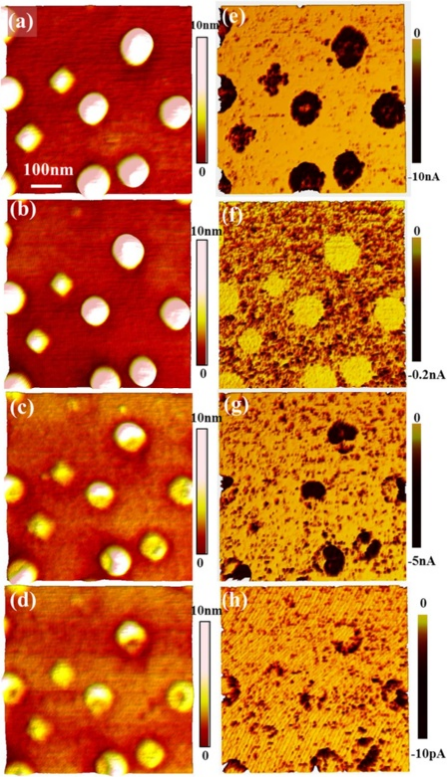
The topography and current distributions of same GeSi QDs before (**a**, **e**) and after NHH etching for totally 2 (**b**, **f**), 7 (**c**, **g**), and 17 min (**d**, **h**), respectively. The sample was biased at −1.0 V and the tip was grounded

On the other hand, the topographic and current images of the same QDs before and after totally 2-, 7-, and 17-min NHH etching measured by CAFM at −1 V are shown in Fig. 2a–h, respectively. Before NHH etching, ring-shaped and cross-shaped current distributions are observed on dome and pyramid QDs, respectively, similar to the results reported in our previous papers [31, 32]. For simplicity, only the dome-shaped QDs will be concerned afterwards, as the result is similar for pyramid-shaped QDs. After the 2-min NHH etching, the remaining GeSi QDs are no longer conductive though the topographic change is slight, which exhibit poorer conductance even more than the WL. However, after the subsequent 5-min NHH etching, the conductance of the QDs is detectable again, exhibiting higher conductance than the WL once again. The QDs after totally 17-min NHH etching still present ring-shaped current distribution, except the current values are greatly reduced. These results are also unexpected, as the QDs after 2-min NHH etching which have higher Ge content exhibit poorer conductance than those after 7- and 17-min NHH etching. To interpret the above inconsistent results, a possible hypothesis is suggested that a thick oxide layer is formed after 2-min NHH etching and still remains on the QDs’ surface after water rinsing.

**Fig. 3:**
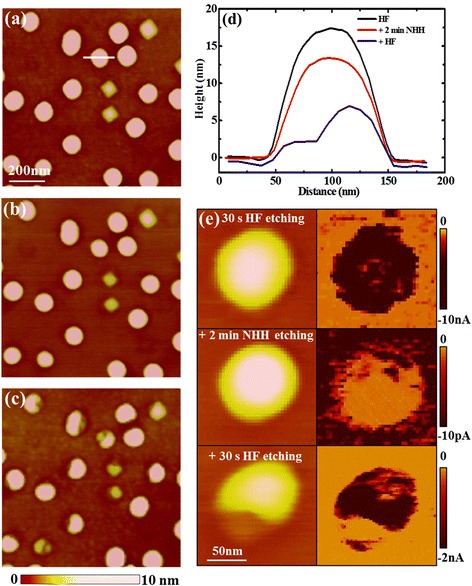
The topography images of the same area after **a** HF etching, **b** HF etching followed by 2-min NHH etching, and **c** further HF dipping added after the NHH etching. The height profiles along the marked line are plotted in (**d**). The topography and current images of a same single QD corresponding to the above three cases measured at −2.0 V are presented in (**e**)

To confirm the above hypothesis, a 30-s HF dipping is added after the 2-min NHH etching. The topography images of same QDs before and after 2-min NHH etching are shown in Fig. 3a, b, while these NHH-etched QDs plus a 30-s HF dipping are shown in Fig. 3c. It can be observed that large parts of the NHH-etched QDs can be etched by HF dipping. The height profiles of a same QD along the same line are plotted in Fig. 3d. It can be seen that an obvious part of the QD, which is about or more than 5 nm in thickness, is etched away by HF dipping. The results therefore confirm that a thick oxide layer is indeed formed and remains on the surface after NHH etching and cannot be removed by water rinsing. The conductive properties of the same QD after different etching processes are also measured, as presented in Fig. 3e. It can be seen that, only after the HF etching, the current can be measured on the QDs’ surface, either without (top) or with (bottom) NHH etching. For the QD only with NHH etching (middle), no current is measured on the QD, even at the lowest current scale of 10 pA.

**Fig. 4:**
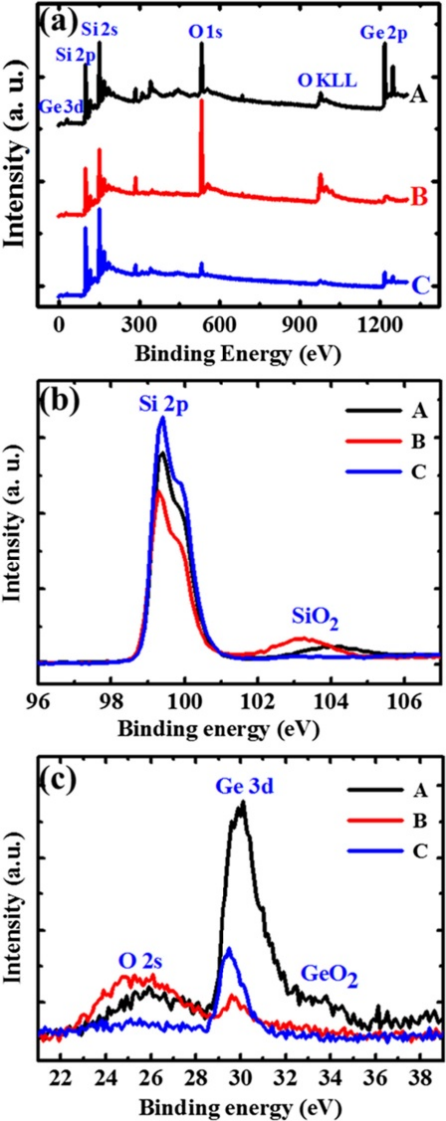
The XPS spectra of the three samples as in Fig. 3 are shown in (**a**). The fine spectra of Si 2p and Ge 3d are given in (**b**) and (**c**), respectively

To further confirm the existence of the oxide layer, X-ray photoelectron spectroscopy (XPS) measurements are performed on the above three samples. The XPS spectra of the same sample with a 30-s HF dipping (termed as sample A), sample A plus a 2-min NHH etching (termed as sample B), and sample B plus another 30 s HF dipping (termed as sample C) are shown in Fig. 4a. As the samples have been exposed to air before transferring into the vacuum chamber, the O 1-s peak can be observed in all the three cases. However, its intensity of sample B is much lager than that of samples A and C, confirming the formation of an oxide layer by NHH etching. The fine XPS spectra of Si 2p and Ge 3d are given in Fig. 4b, c, respectively. One can observe that the Si oxide signal of sample B is obviously larger than that of samples A and C, while almost no Ge oxide signal is measured on sample B together with the large decrease of the Ge 3d signal. Thus, the XPS results confirm that a thick oxide layer is formed in the NHH etching and it is Si oxide.

Based on the above results, it can be demonstrated that a thick oxide layer is formed and remains on highly GeSi mixed QDs after NHH etching. Particularly, the above phenomena can only be observed on highly GeSi mixed QDs (e.g., grown at 680 °C). Similar measurements have been performed on both GeSi QDs grown at 570 °C and 640 °C which contain higher Ge contents. Normal composition distribution and conductive behaviors are measured, where the conductance monotonically decreases with the etching time (decreased Ge content), indicating the oxide layer formed in those cases is much thinner. On the other hand, from the above result as shown in Fig. 2f, it can seen that the conductance of the WL is better than that of the QDs, suggesting that the oxide layer formed on the WL, which has higher Si content, is thinner than that on the QDs. Consistently, the oxide layer formed in the subsequent 5-min NHH etching which is done with a pretreatment of HF dipping is much thinner than in the former 2-min etching, as the current can be detected again after this etching process. These results suggest that the oxide layer thickness decreases with the Ge content decreasing. Therefore, the formed oxide layer thickness is obviously related to the Ge content, and it is large for highly GeSi mixed QDs. By either increasing or decreasing the Ge content, the thickness of the formed oxide layer by NHH etching decreases.

The origin why the formed oxide layer thickness varies with Ge content is not clear yet, and only a rough assumption is supposed as follows. As stated in previous literatures [20, 21, 34], Ge can be removed in aqueous H_2_O_2_ solutions since it is oxidized by the latter and its oxide is water soluble, while Si can be removed by NH_4_OH solution but the etching rate decreases with the addition of H_2_O_2_. When NHH solution etches GeSi alloys with high Ge contents, the formed oxide is mainly Ge oxide which can be dissolved in water, so no or little oxide layer would remain on the QDs’ surface. Contrary to the Ge oxide, Si oxide is much stable and dissolved slowly in NHH solution. But the fast passivation of Si by the Si oxide would stop the etching procedure. Thus, etching of GeSi QDs with high Si contents would also result in a thin Si oxide layer on the QDs’ surface and hence the current is still detectable. On the contrary, when NHH etches highly GeSi mixed QDs (medium Ge content of 40–60 %), the presence of Ge or the GeSi mixing can significantly enhance the oxidation of Si without passivation. In previous researches dealing with thermal oxidation of GeSi alloys [35, 36], it was indeed found that Ge could act as a catalyst during oxidation of Si, which was interpreted by the reduced binding energy of Si atoms at the interface due to alloying with Ge [35]. While the oxidation of Si is enhanced by alloying with Ge, the passivation of Si by the Si oxide is reduced by the presence of Ge contrary. Therefore, the stopping of Si oxidation is impeded; as a result, a thick Si oxide layer is formed on highly GeSi mixed surface.

**Fig. 5:**
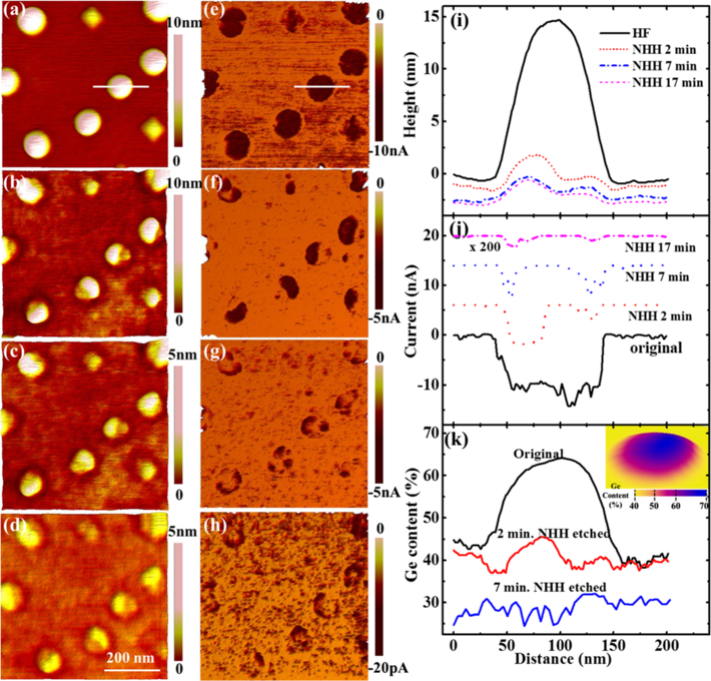
The topography and current images of the same QDs before (**a**, **e**) and after NHH etching for totally 2 (**b**, **f**), 7 (**c**, **g**), and 17 min (**d**, **h**), respectively. Here, a 30-s HF dipping is added after each NHH etching. The height and current profiles along the marked line before and after each etching process are shown in (**i**) and (**j**), and the deduced composition profiles from (**i**) are given in (**k**). The surface composition distribution of the marked QD is shown as the inset in (**k**)

Since an oxide layer would be formed and remain on the QDs’ surface as well as on the WL which are not taken into the calculation of etching rate, the formerly obtained composition and current distributions are no longer correct. Therefore, to get the exact etching rate and hence the composition distribution, a 30-s HF dipping is added after each NHH etching. The topography and current images of the samples measured similar as Fig. 1 are shown in Fig. 5. With the method mentioned above, the GeSi composition distributions of both QDs and WL can be obtained. The line profiles of the height and current along the same marked line on the same QD before and after each etching process are plotted in Fig. 5i, j, respectively. The Ge content profiles deduced from the etching rate along the same line is given in Fig. 5k. The etched height of the WL is about 0.7, 1.0, and 0.4 nm for the first 2-min and subsequent 5- and 10-min NHH etching, respectively, giving the Ge content of about 45 % for original WL surface and about 40 and 30 % for 2- and 7-min NHH-etched WL surface, respectively. For the QD, the etched height after the initial 2-min NHH etching is up to 14 nm at its right center and about 12 nm at its left center, suggesting that the high Ge content (~65 % at right center and 60 % at the left center) is concentrated on its top center. The Ge content decreases from top to bottom and from center to edge. The Ge content is almost uniform on the 7-min NHH-etched surface. The surface composition distribution of the marked QD is also obtained by subtracting the same QD’s topography after etching from that before etching and converting the etched height distribution to composition distribution, as presented in the inset of Fig. 5k, showing a similar top-centered Ge-rich distribution.

On the other hand, from the current profiles as shown in Fig. 5j, similar ring-shaped distributions are observed for all dome-shaped QDs, except some QDs present asymmetric distributions corresponding to their asymmetric topographic shape. The ring-shaped feature is not very obvious for the QDs before NHH etching, which is most probably due to the saturation effect of the current at the QDs’ edges. After NHH etching, the composition distribution of QDs greatly changes, but the ring-shaped current distribution as well as dome-shaped topography remains, except the dome height and current values greatly decrease with the decreasing of Ge content. The origin of the ring-shaped current distribution has been interpreted in our previous paper [31] as follows: a large number of Si atoms in the substrate were incorporated into the Ge QDs at the high deposition temperature, forming roughly uniform GeSi alloys in the QDs with high Si concentration, resulting in large dot resistance. As the dot resistance is proportional to the current path length, i.e., dot height, it has a smaller value at its side region than at its center. Furthermore, the contact area between the tip and dot surface is also larger at the periphery, forming ring-shaped current distribution. Since the dome-shaped QDs with different GeSi compositions (after different etching processes) exhibit similar ring-shaped current distribution, it can de declared that the QDs’ current distribution is mainly determined by its topography, while the current values are greatly influenced by the Ge content, similar to the conclusion obtained on GeSi quantum rings [33, 37].

## Conclusions

In conclusion, the conductive properties and the composition distributions are obtained simultaneously on same single QDs by CAFM measurements combined with NHH etching with a nanoscale trench. In particular, it should be noticed that, for highly GeSi mixed QDs grown at 680 °C, the NHH etching would form a thick oxide layer on the QDs’ surface, resulting in poorer conductance even more than the WL. This finding should be useful for similar etching experiments, as it has never been concerned previously. By adding a HF dipping after each NHH etching, the exact composition and current distributions as well as their correlation are obtained. The results suggested that the ring-shaped current distribution is determined by its topography while the composition distribution mainly influences the current values.
